# Perceived risk of COVID-19 pandemic on crop production: an implication for food security in Ethiopia

**DOI:** 10.1186/s12889-023-15677-w

**Published:** 2023-04-24

**Authors:** Daba Ejara, Megersso Urgessa, Kebede kumsa, Junayde Abdurahaman

**Affiliations:** 1 Department of Nursing, School of Health Sciences, Madda Walabu University, Shashemene campus, Robe, Ethiopia; 2 Department of Public Health, School of Health Sciences, Madda Walabu University, Shashemene campus, Robe, Ethiopia

**Keywords:** COVID-19, Food security, Risk perception, Crop production

## Abstract

**Background:**

Risk perception is a subjective psychological construct that is influenced by cognitive, emotional, social, cultural, and individual differences, both within and between individuals and across countries. Although the impact of COVID-19 on short- and long-term food security is difficult to predict, some risk factors and lessons from previous pandemics can be identified. The goal of this study is to assess rural farmers’ perceptions of the COVID-19 pandemic’s impact on crop production and its implications for food security in West Arsi Zone, Oromia, Ethiopia.

**Methods:**

A community-based cross-sectional study was conducted among 634 small-holder farmers in the west Arsi zone district. From November 1–30, 2020, data was gathered through interviews with local farmers. Data was gathered using a semi-structured questionnaire. Six trained expert agricultural workers were used as data collectors and supervisors, respectively, and both were trained. The questionnaire had been pre-tested. The Statistical Package for the Social Sciences (SPSS) software version 25 was used to analyze the data. To identify factors associated with risk perception of the COVID-19 pandemic on crop production, binary and multivariable logistic regression were used, with statistical significance determined at a p-value of 0.05.

**Results:**

This study found that among farmers in West Arsi Zone, Oromia, Ethiopia, about 32.5% reported having perceived risk of COVID-19 pandemic on crop production, and that age greater than or equal to 57, female sex (AOR,1.48 95% CI (1.03–2.12)), primary Educational status (AOR,2.85(1.78–4.58)), and permanent employed occupation of the house head (AOR, 2.27(1.24–4.17) were found to be independent predictors of perceived risk of COVID-19 pandemic on crop production among farmers in West Arsi Zone, Oromia, Ethiopia.

**Conclusion:**

Perceived risk of COVID-19 on crop production was high and varied across age groups, sexes, educational attainment levels, and the occupation of the head of the household.

## Introduction

The coronavirus disease (COVID-19), an infectious disease brought on by the coronavirus, has been ravaging lives and livelihoods since late 2019 [1]. The virus was first discovered in Wuhan City, and within a few months, it had spread to every country on earth [2]. Therapeutics and vaccines are not yet readily available. The virus has caused significant, abrupt changes in the global healthcare, economic, transportation, agricultural, and educational systems [3].

Agriculture unquestionably contributes significantly to economic development and growth in every nation in the world [4]. Similar to this, agriculture is the largest industry and the primary source of income for most people worldwide, especially in developing nations [5–7]. While the Corona virus is a catastrophe for public health, questions are raised about its potential effects on local and global food systems and their ability to ensure access to and consumption of safe and affordable food as well as adequate incomes for those living, in particular, in the smallholder sector of developing countries [3]. Even though it is difficult to predict how COVID-19 will affect short- and long-term food security, some risk factors can be identified, and lessons learned from past pandemics (such as the Ebola virus disease (EVD) in West Africa in 2014) or major crises (such as the food prices crisis of 2008) suggest that effects on food security could be swift and of dramatic proportions [1].

Previous epidemics, such as SARS and H7N9, have had devastating effects on population health, crop production, and the economy [8]. COVID-19 outbreaks could be much more difficult to control in countries already affected by existing shocks, such as political instability and conflict, and could potentially exacerbate food insecurity [1]. As Ethiopia is currently affected by desert locust outbreaks or economic crises, an additional layer of COVID-19 impacts has been added to the challenges that these areas are already facing, and is likely to increase the number of vulnerable people. Because of access constraints and health-care capacity limitations, the disease could spread much faster and have more severe consequences, making it difficult to diagnose and contain. Food supply chain disruptions and a decrease in access to basic necessities could lead to an increase in food insecure households. Price increases, economic insecurity, and fear as the pandemic spreads could all contribute to increased social tension.

Crop production inputs such as fertilizer and improved seeds, as well as judicious pesticide use and improved agronomic practices, are required to increase agricultural production and productivity. Fertilizer and high yielding crop varieties are the most important crop production technologies in Ethiopia. To obtain agricultural inputs, smallholder farmers rely on primary cooperatives, cooperative unions, and, most importantly, informal markets [9]. Lockdowns caused by the pandemic in input producing countries could disrupt timely delivery/transport of inputs, negatively impacting crop production.

Ethiopian farmers may not only have their production and incomes disrupted, but may also have challenges in accessing food for personal consumption. As noted by Arif Husain, the World Food Programmer’s Chief Economist, ‘countries with high levels of food insecurity and high levels of poverty are generally more vulnerable and less prepared for an pandemics and would likely see higher mortality rates’ [10].

To control its effect of COVID-19 pandemic the government of Ethiopia has tried to mitigate the effect and build resilience; the government has given more financial support to health care, and financial sectors and tax exemptions of imported products for the prevention and containment of COVID-19. However, there can be food security and agricultural risks coming from the COVID-19 crisis unless clear advices and decisions for mitigations are outlined [11].

Therefore, there is a need for careful planning in food supply considering the various actors along the food value chain and mainly on an inclusive basis, considering the vast, vulnerable groups of the residents of the zone to food insecurity. So that the objective of this study is early assessing perceived risk of COVID-19 pandemic on crop production and its implication for food security among rural farmers in West Arsi Zone, Oromia, Ethiopia.

## Methods

### Study participants

A Community base cross-sectional study was conducted in West Arsi Zone, Oromia regional state, Ethiopia. Farmer households who were actively involved in agricultural production were included for this study using Systematic sampling technique. Whereas Farmer households who were retired and not involved in agricultural production were excluded from study. The sample size of the study was determined by using for single population proportion formula with assumptions of 50% prevalence, 95% confidence level, 5% degree of desired precision or margin of error for sampling, a design effect of 1.5; since we use multistage sampling technique and 10% for non-response rate. (n = sample size, p = prevalence, w = margin of error).

$$n = \frac{{[{{\left( {{Z_{\alpha /2}}} \right)}^2}*{\rm{ }}\left( {p{\rm{ }}\left( {1 - p} \right)} \right]}}{{{w^2}}}$$

$$n = \frac{{{\rm{[(1}}{\rm{.96)2 * 0}}{\rm{.5(1 - 0}}{\rm{.5)]}}}}{{{{\left( {0.05} \right)}^2}}} = 384$$

So the sample size will be multiplied by design effect of 1.5, **n** = 384 * 1.5 = 576 and adjustment for non-response (10% contingency) **n**= (576 + 58) = 634 farmers.

**Table:** Study Variable

Dependent variable	Risk Perception on COVID-19
**Independent variables**	1-Socio-demographic characteristics Age of respondents sex Educational status. Marital status
	2- Implications of COVID-19 on the supply chains functions and stages, - Production - Handling and storage - Processing and packaging Distribution and marketing and Consumption level

### Data collection tool

Data were collected by face-to-face interviewer administered questionnaire by using a structured questionnaire which prepared in English language then translated to local language a pretested. Ethical clearance was obtained from ethical review board of Madda Walabu University and official letter was written to each kebele administrative office for ethical approval and permission. Verbal and written Consent was taken from study subjects. Confidentiality of collected information was also ensured.

### Data analysis

Data were checked for completeness, code and consistency for data entry and cleaning; then data were entered into the Epi-info version 7.2.1 and exported to SPSS version 23 for analysis. Descriptive statistics, using frequency table, proportions, mean, standard deviation, and cross tabulations will use to present the study results. Normality of all continuous variables were checked by using the Kolmogorov–Smirnov test at p value > 0.05. Multi-co-linearity of variables will be checked be using Variance Inflation Factor (VIF) at p-values less than 10%. Hosmer-Lemeshow χ2 test was used to assess the goodness-of fit of the models at p-values of greater than 0.05.

Binary logistic regression was done to determine the associations between each independent variable and outcome variable; and variable with p < 0.25 level of significant in bi-variable analysis was moved to multivariable logistic regression considering p value < 0.05 and finally, adjusted OR/RR with 95% confidence interval to determine the independent association on perceived risk of crop production and interaction between predictor variable at p value < 0.05.

## Results

### Socio demographic characteristics of study subjects

In this study from 634 respondents were participated in the study giving the response rate of 100%. Out of 634 participants more than half (51.4%) were Female. About 89.5% of study subjects were between the ages 16–46 years (Table 1).

**Table 1:** Socio demographic characteristic of respondents, west Arsi zone, Ethiopia, 2020. (n = 634)

Characteristics	No.	Percent
**Age of respondents**		
16–26	171	27
27–36	259	40.9
37–46	138	21.8
47–56	44	6.9
>=57	22	3.5
**Sex of respondents**		
male	308	48.6
Female	326	51.4
**Ethnicity**		
oromo	594	93.7
Amahara	22	3.5
Gurage	8	1.3
Wolayita and other	10	1.57
**Address**		
urban	463	73
Rural	171	27
**Marital Status of the respondent**		
Single	36	5.7
Married	44	6.9
Separated/divorced	538	84.9
Widowed	16	2.5
**Education status of House Hold head**		
No education	61	9.6
Primary(1–8)	192	30.3
Secondary (9–12)	158	24.9
diploma/Degree	223	35.2
**Educational level of wife**		
No formal education	132	20.8
Primary school(1–8)	233	36.8
Secondary school(9–12)	152	24
Diploma /Degree and above	117	18.4
**Occupation status of household head**		
Permanent employee	173	27.3
Temporary employee	32	5
No work	19	3
Pension	151	23.8
Farmer	259	40.9
**Religion of the farmer**		
Muslim	500	78.9
orthodox	55	8.7
Protestant	74	11.7
other	5	0.8
**Family size**		
< 5 member	391	61.7
> 5 member	243	38.3
**Land owner ship in hector**		
no land	282	44.5
< 2 hector	276	43.5
2–4 hector	43	6.8
>= 4 hector	33	5.2
**Owner ship of house**		
no	146	23
yes	488	77
**water source**		
protected( pipe, bono)	570	89.9
Not protected (spring, river)	64	10.1
**Wealth index quintile**		
Lowest	2	0.3
Second	120	18.9
Middle	462	72.7
Fourth	47	7.7
Highest	1	0.2

### Risk perception of the residents due COVID-19 pandemic

From all 634 respondents interviewed 597(94.2%) where worried personally about COVID-19, and also 570 (89.9%) of the study participant fear that they likely personally affected by Catching the COVID-19 (Table 2).

**Table 2:** Showing Risk Perception of the Residents due COVID-19 Pandemic in West Arsi Zone 2020

**Do you agree that COVID-19 will not affect very many people our country**		
Agree	337	53.2
Disagree	297	46.8
**Do you agree that you probably get sick with COVID-19**		
Agree	264	41.6
Disagree	370	58.4
**Do you agree getting sick with COVID-19 can be serious?**		
Agree	337	53.2
Disagree	297	46.8
**To what extent you think farmers have good understanding of COVID − 19**		
Have understanding	160	25.2
Not have understanding	474	74.8
**Do you perceive risk of getting infected by COVID-19**		
not all at risk of getting infected	81	12.8
have at risk of getting infected	553	87.2
**Do you perceive risk of having serious illness by COVID-19**		
not at all likely,	68	10.7
likely risk of serious illness	566	89.3
**Do you perceive risk of deaths by COVID − 19**		
not at all likely,	68	10.7
likely risk of death	566	89.3

### Perceived risk of COVID-19 pandemic on crop production

Perceived Risk of COVID-19 Pandemic on Crop Production varies among respondents based on components with highest Impact of COVID-19 on availability of human power607(95.7%) and lowest Effect on handling and storage of agricultural products 575(90.7%) (Table 3).

**Table 3:** Showing Perceived Risk of COVID-19 Pandemic on Crop Production in West Arsi Zone 2020

Risk of COVID-19 Pandemic on Crop Production		
	Yes	No
Effect of COVID-19 on improved seed supply	600(94.6%)	34(5.4%)
Perceived impact of COVID-19 on fertilizer supply	597(94.2%)	37(5.8%)
Impact of COVID-19 on availability of human power	607(95.7%)	27(4.3%)
Impact of COVID-19 on labor cost.	593(93.5%)	41(6.5%)
Effect of COVID-19 on availability of pesticide	598(94.3%)	36(5.7%)
Effect on handling and storage of agricultural products	575(90.7%)	59(9.3%)
Effect on processing and packaging of production	599(94.5%)	35(5.5%)
Effect on distribution and marketing of production	606(95.6%)	28(4.4%)
Effect on food consumption level in your house hold	585(92.3%)	49(7.7%)

### Perceived risk of COVID- 19 pandemic on crop production and associated factors

Crop production of age group (47–56) was found to be 10 times more Perceived Risk of COVID- 19 Pandemic on Crop Production than other age groups AOR 10.13(2.96–34.6) p-value 0.000 and also as age increase near to old the Perceived Risk of COVID- 19 Pandemic on Crop Production was increased. In addition, Perceived Risk of COVID- 19 Pandemic on Crop Production among Diploma / Degree and above education status were 2 times perceived as compared to other level with AOR 1.83(1.07–3.15) p-value 0.003( Table 4).

**Table 4:** Binary and Multivariate Logistic Regressions of Factors Associated with Perceived Risk of COVID- 19 Pandemic on Crop Production in West Arsi Zone, Southern Ethiopia

Variable	No (%)	COR(95% CI)	AOR(95% CI)	p-value
**Age of the respondent**				
16–26	171	7.17(2.52–20.4)	5.01(1.7–14.8)	0.003
27–36	259	7.21(2.57–20.21)	5.78(2.03–16.45)	0.001
37–46	138	8.04(2.78–23.3)	6.97(2.39–20.32)	0.000
47–56	44	11.6(3.41–39.2)*	10.13(2.96–34.6)*	0.000*
>=57	22	1	1	
**Sex of the respondent**				
Male	308	1	1	
Female	326	1.54(1.095, 2.15)*	1.48(1.03–2.12)	0.03*
**Residence of the respondent**				
urban	463	1		
Rural	171	0.73(0.50, 1.05)	0.72(0.49–1.06)	0.09
**Marital status of the respondent**				
Single	36	1	1	
Married	44	1.81(0.72, 4.52)	2.16(0.84–5.57)	0.11
Separated/divorced	538	1.96(0.98, 3.91)	1.91(0.95–3.83)	0.07
Widowed	16	0.66(0.19, 2.16)	1.11(0.32–3.89)	0.87
**Educational status of house hold head**				
No education	61	1	1	
Primary(1–8)	192	4.11(2.25–7.54)*	3.58(1.87–6.86)	0.00**
Secondary ( 9–12)	158	2.43(1.33–4.43)	2.21(1.17–4.21)	0.02
(diploma/Degree)	223	2.59(1.45–4.61)	2.29(1.21–4.34)	0.01
**Educational level of wife**				
No formal education	132	1	1	
Primary school(1–8)	233	3.11(1.99, 4.88)*	2.85(1.78–4.58)*	0.00**
Secondary school(9–12)	152	4.41(2.62, 7.43)	4.19(2.39–7.33)	0.00
Diploma / Degree and above	117	1.94(1.17, 3.23)	1.83(1.07–3.15)	0.03
**Occupation of the house head**				
Permanent employed	190	1	1	
Un employed	88	1.89(1.06, 3.39)*	2.27(1.24–4.17)*	0.01
Farmer	259	1.14(0.77, 1.69)	1.21(0.79–1.85)	0.38
Merchant	97	1.08(0.65, 1.81)	1.09(0.64–1.86)	0.75
**Family size**				
Less than 5	391	1	1	
Greater than 5	243	0.94(0.67, 1.32)	0.84(0.58–1.22)	0.36
**Land owner ship**				
**no land**	282	1	1	
< 2hector	276	0.94(0.66–1.34)	1.02(0.68–1.52)	0.93
>=2 hector	76	0.68(0.41–1.16)	0.75(0.43–1.30)	0.31

## Discussion

Globally, COVID-19 pandemic created health and human crisis threatening the food security and nutrition of many people. The combined effects of COVID-19 itself, as well as corresponding prevention measures and the developing global failure could, without large-scale coordinated action, disrupt the functioning of food systems. Such disruption can result in consequences for health and nutrition of a severity which is directly linked with crop production. The aim of this study were to assess perceived risk of COVID-19 pandemic on crop production and its implication for food security among rural farmers in West Arsi Zone.

Based on this we assessed Socio-demographic characteristics of the respondents, perceived risk of COVID-19 on the supply chains functions and stages such as, Production, Handling, Processing/ packaging, Distribution and marketing. The finding of Bivariate and Multivariate logistic regression showed that age is greater than or equal to 57, female sex, primary Educational status, Occupation of the house head found statically significant association with perceived risk of COVID-19 on crop production.

This study shows that about 32.5% people reported having Perceived risk of COVID-19 pandemic on crop production and Perceived Risk of COVID-19 Pandemic on Crop Production is affected by age of Study participants, among respondents of different age group as age increase their is increased risk of failure on crop production. Crop production of age group (47–56) is found to be 10 times more at risk than other age groups AOR 10.13(2.96–34.6) p-value 0.000 and also as age increase near to old the risk increase consequently. This finding is consistent with the study done in Gondar City northwest Ethiopia reported Study participants with age greater than 45 years had 1.41 times risk of experiencing high perceived risk towards COVID-19 than participants ageless than 26 years old. being older is associated with a higher chance of adopting preventive behavior during pandemic due to greater level of susceptibility and perceived severity of disease [12]. This is possibly due limited movement to do certain activity because of fear of COVID − 19 infection and they are also older age were less active group in crop production activity in the society. They are near to dependency age group. This indication helps to identify the target group for the intervention.

Regarding female respondents, Perceived Risk of COVID-19 Pandemic on Crop Production were 1.48 times higher when compared with male respondents AOR 1.48(1.03–2.12),p-value 0.03. This finding is supported with the study done in Nigeria by [13] reported during COVID-19 pandemic Male headed households are more food secure compared to their female headed household counterparts [14] This may be because in our country particularly in west Arsi zone case male are more economically dominant than female. As well most female stay at home and they have low participation in crop production activity. Beside this female are less educated and low chance of being landownership than male which directly affects their means of income generation such as crop production, storage, packing, distribution and consumption this in turn ultimately affects food security.

Perceived Risk of COVID-19 Pandemic on Crop Production is higher among house hold head whose educational status is 1–8 when compared with those individual do not have no education. 3.58(1.87–6.86) p-value < 0.001, this is possibly because of this secondary and above education status may have capacity to use better approach and technology for crop production. this finding is in line with the study done in Gonder reported Participants with college and above educational status had 72% lower perceived high risk for COVID-19 compared with participants with no formal education [12]. The possible explanation for this might be low educated individuals not effectively use modern technology so they do not have sufficient information how to overcome challenges occurred due to COVID − 19 prevention measure such as restrictions and lockdown and participants with high education background has a better understanding to apply the precaution measures to prevent the risk of the pandemic so that they lower perceived risk for the pandemic on agricultural production.

Concerning Occupation of the house hold head, un-employed respondents were 2.3 times increased perceived Risk of COVID-19 Pandemic on Crop Production compared with Permanently employed individuals AOR,2.27(1.24–4.17) p-value 0.01. This findings were comparatively similar with study done in Iran [15]. Another study done in Kenya and Uganda showed that the income-poor households and those dependent on labor income were more vulnerable to income shock, and had poorer food consumption during the COVID-19 pandemic compared to other respondent categories [16]. This may be due to the fact that unemployed individuals who have low income were less effective in implementing prevention method as well they give priority to means of getting food. Moreover, staying at home is difficult for them without having food since they are unemployed even though they have knowledge about COVID-19 prevention measure.

## Conclusion

The study’s goal is to assess rural farmers’ perceptions of the COVID-19 pandemic’s impact on crop production and its implications for food security in West Arsi Zone, we conducted Community base cross-sectional study and the study showed there is a high risk perception. Risk perception varies with minimal differences across different categories.

Respondent’s age, sex, Educational status, and occupational status of household head is the determinants of Perceived risk perception of COVID-19 on crop production.

We recommend increasing the provision of an appropriate environment and educating on adequate preventive measures by organizing campaigns for individuals, regardless of social status, to follow the rules to prevent COVID-19. Social support policies, in addition to financial assistance, should be implemented for vulnerable populations.

## Data Availability

All data generated or analyzed for this study are available from the corresponding authors upon reasonable request.

## Abbreviations

COVID- 19: corona virus disease 2019.
EVD: Ebola virus disease
FAO: Food and Agriculture Organization of the United Nations ()
H7N9: Avian Influenza A(H7N9) virus
SARS: severe acute respiratory syndrome
SARS: Severe Acute Respiratory Syndrome and
MERS: Middle East Respiratory Syndrome
VIF: Variance Inflation Factor
